# Adaptation and Dissemination of a National Cancer Institute HPV Vaccine Evidence-Based Cancer Control Program to the Social Media Messaging Environment

**DOI:** 10.3389/fdgth.2022.819228

**Published:** 2022-07-27

**Authors:** Suellen Hopfer, Kalani Kieu-Diem Phillips, Maxwell Weinzierl, Hannah E. Vasquez, Sarah Alkhatib, Sanda M. Harabagiu

**Affiliations:** ^1^Program in Public Health, Department of Health, Society, and Behavior, University of California, Irvine, Irvine, CA, United States; ^2^Department of Computer Science, University of Texas at Dallas, Richardson, TX, United States

**Keywords:** HPV, HPV vaccine, social media, implementation adaptation, engagement, vaccine interventions

## Abstract

Social media offers a unique opportunity to widely disseminate HPV vaccine messaging to reach youth and parents, given the information channel has become mainstream with 330 million monthly users in the United States and 4.2 billion users worldwide. Yet, a gap remains on how to adapt evidence-based vaccine interventions for the *in vivo* competitive social media messaging environment and what strategies to employ to make vaccine messages go viral. Push-pull and RE-AIM dissemination frameworks guided our adaptation of a National Cancer Institute video-based HPV vaccine cancer control program, the HPV Vaccine Decision Narratives, for the social media environment. We also aimed to understand how dissemination might differ across three platforms, namely Instagram, TikTok, and Twitter, to increase reach and engagement. Centering theory and a question-answer framework guided the adaptation process of segmenting vaccine decision story videos into shorter coherent segments for social media. Twelve strategies were implemented over 4 months to build a following and disseminate the intervention. The evaluation showed that all platforms increased following, but Instagram and TikTok outperformed Twitter on impressions, followers, engagement, and reach metrics. Although TikTok increased reach the most (unique accounts that viewed content), Instagram increased followers, engagement, and impressions the most. For Instagram, the top performer, six of 12 strategies contributed to increasing reach, including the use of videos, more than 11 hashtags, COVID-19 hashtags, mentions, and follow-for-follow strategies. This observational social media study identified dissemination strategies that significantly increased the reach of vaccine messages in a real-world competitive social media messaging environment. Engagement presented greater challenges. Results inform the planning and adaptation considerations necessary for transforming public health HPV vaccine interventions for social media environments, with unique considerations depending on the platform.

## Introduction

Social media has become a mainstream health information channel with 330 million monthly users in the United States (1). Consequently, it has rapidly become a crucial public health communication tool for information dissemination and consumption (2). Social media channels offer a key opportunity for implementing and sharing accurate, timely, and culturally resonant public health messages, including vaccine messaging. However, a significant gap remains in how to effectively adapt evidence-based interventions (EBI), especially for the social media environment. We define adaptation as a systematically planned and proactive process of intervention modification to suit the specific characteristics and needs of a new context and enhance intervention acceptability (3). Dissemination of public health messages in social media environments requires a paradigm shift in dissemination approaches that departs from one-directional hypodermic needle dissemination (4) to a dialog-based, push-pull dissemination approach (5). We address a gap in the dissemination of EBI. Our 4-month observational social media study describes the intervention adaptation processes necessary for disseminating narrative human papillomavirus (HPV) vaccine intervention across three platforms in the real-time social media messaging environment (6).

The rise of social media during the digital age has fundamentally changed how individuals seek and receive health information (7). In the United States, 84% of 18- to 29-year-olds report using social media, with 71% reporting daily Instagram use and 65% reporting daily TikTok use (7). Other popular social media platforms among young adults include Reddit, Twitter, and YouTube (7). Each platform has different ways of sharing information, but all are predominantly peer-driven and user amplified (8). The use of social media for seeking health information, including vaccine information, was amplified during the COVID-19 pandemic (2). Given the number of social media users worldwide, these platforms offer a dynamic communication channel for disseminating preventive health messages and expanding reach to historically underserved populations (9). Disseminating accurate, yet culturally resonant vaccine information is critical in light of the polarized social media environment, particularly around vaccination (10–12).

Identifying effective social media strategies to increase HPV vaccination rates is a priority for the Centers for Disease Control and Prevention (CDC), World Health Organization, American Cancer Society, National HPV Vaccination Roundtable, and all National Cancer Center (NCI)-designated cancer centers in the United States (13–16). National roundtable experts prioritize the adaptation and dissemination of social media strategies to increase awareness and reach among unvaccinated youth, young adults, and parents (17). Yet, limited guidance exists on how to implement and adapt evidence-based HPV vaccine interventions on social media (18, 19). Drawing on an implementation and dissemination scientific framework, the push-pull capacity model (5, 20), and RE-AIM (19, 21), our observational study aimed to understand how the social affordances of social media can be harnessed to effectively disseminate an evidence-based HPV vaccine intervention.

The push-pull capacity dissemination framework (5, 20, 22) applied to the social media messaging environment includes a “push” intervention component to broadcast vaccine messages to media users and a “pull” component to engage users with intervention content, invite comments, encourage interaction, and share content with peers. Pushing our HPV vaccine content on social media is expected to evoke curiosity and interest in HPV vaccination, and generate more impressions on our posted content, while the pull component, which is whether and how various adaptation strategies may engage users, remained an impetus for the study.

The RE-AIM implementation framework (19, 21) that also guided our study proposes that the success of adapting an intervention is best evaluated by five dimensions: reach, effectiveness, adoption, implementation, and maintenance. In our study, we sought to evaluate the first of the five dimensions, to examine the reach of broadcasting the HPV vaccine narratives across three platforms: Instagram, TikTok, and Twitter.

### Social Media Platforms Used by Youth and Young Adults

Instagram, founded in 2010 and one of the most popular social media platforms, caters to 500 million daily active users and 1 billion monthly active users worldwide (1). Among all users, 7.5% are 13–17, 29.9% are 18–24, and 32.1% are 25–34 years old (1). Thus, nearly one-third of active users are in the target age group (13–24 years old) for HPV vaccine promotion messages. Moreover, Instagram information sharing largely centers on videos, photos, and stories (23), which matches the format of our HPV vaccine videos and narratives.

TikTok, founded in 2017 and a video-centric global social media platform used by youth, has more than 1 billion monthly active global users and an estimated 80 million monthly users in the United States. The platform allows users to create and share short videos and has become the most downloaded non-gaming app globally (24). The user interface design facilitates easy-to-use editing and soundtrack functionality. TikTok's feed is unique compared to other platforms; users can interact with the “just for you” page and #Discover (by hashtag or audio file), which the TikTok algorithm populates with videos that have a high likelihood of user engagement. Engagement differs from YouTube where video length has no limit and users have more autonomy in choosing the next video to watch. Nearly half (47.4%) of the billion monthly users are 9–26 years old (25). TikTok's platform features 15-s video streams—with a recent update to post videos up to 3 minutes (26)—which are typically entertainment-based. The platform is distinct from Instagram in the sense that it does not allow the posting of photographs or infographics. TikTok is increasingly considered for disseminating health information, especially during the pandemic (27). HPV vaccination is recommended for preteens aged 9–11, and catch-up vaccination is recommended for young adults up to age 26 (28). The focus of this cancer control program is HPV vaccination and since TikTok has a large portion of users in our target population, we chose to include this platform.

Twitter, founded in 2006 and is text-based, is public and has 206 million daily active users, 38.5% of which are in the target age group for our HPV vaccine promotional messages (29). Unless privacy is specifically chosen by the user, Twitter posts, profiles, and materials are all automatically public, and hence, the data is publicly available (30). Although Twitter has the capability of posting photos and videos, the platform is predominantly text-based, fostering text interactions among users and conversation threads (31).

We chose these three social media platforms for their unique properties and potential to propagate health information to reach youth and young adults. We chose Instagram and TikTok due to the number of Millennial and Gen Z users on these platforms (1). Millennial users are defined as individuals born between 1981 and 1994 (32), while Gen Z users are born between 1995 and 2015. Additionally, due to the algorithm and functionality of Instagram and TikTok, there is more potential for users of these platforms to watch HPV videos, especially if any of them went “viral” (33). This is because “reels” posted from a public account can be viewed by anyone and not just network users on the reels tab on Instagram. Similarly, for TikTok, anyone can view a public video posted by a public account on their “for you” page (34). Using multiple strategies, we were interested to learn which strategies would propagate our vaccine messages the most and how performance differed by platform.

### HPV Vaccination as a Highly Effective Cancer Prevention Measure

The HPV vaccination, federally licensed in 2006, is highly effective at preventing high-grade and persistent cervical intraepithelial neoplasia in women and is more than 92% effective at preventing HPV-associated cancers, including cervical, anal, and oropharyngeal cancers, with the latter in both men and women (35, 36). An estimated 14 million Americans are infected with new HPV cases annually as the most common sexually transmitted infection in the United States. More than half of these infections occur in people younger than 24 (37, 38). HPV is associated with 2 million HPV-attributable abnormal Pap smears, 1.4 million low-grade cervical dysplasias, and 300,000 high-grade cervical dysplasias annually (16). Yet, despite the robust safety profile of HPV vaccination that has reduced HPV morbidity by 64% among vaccinated 14- to 19-year-olds (39, 40), low vaccination rates (55%) persist particularly among young adults aged 18–26 and have dropped drastically during the pandemic. The CDC's Advisory Committee on Immunization Practices and all US cancer centers recommend urgent catch-up vaccination (16).

### Adapting an NCI Evidence-Based HPV Vaccine Cancer Control Program

To disseminate the evidence-based NCI cancer control HPV vaccine program (EBCCP) HPV Stories (41), the video-based intervention was adapted for the social media environment to reach youth, young adults, and parents of adolescents. The program consists of five parent videos and 13 young adult videos. The 1- to 2-minutes videos each present vaccine decision narratives told by youth through informal conversation. Videos were filmed from the perspective of a voyeur witnessing a private dialog between two individuals (mother-daughter, peers, or romantic partners) in informal settings (e.g., in the kitchen, in a parked car, on a park bench, on the sofa while gaming, on the front steps of a brownstone, at a pool, and at a salon). Some videos also reflect conversations between doctors and families in a clinical setting in an exam room.

We describe the adaptation process of implementing a 4-month observational study by posting 13 HPV vaccine decision story videos on an HPV vaccine account. A second aim of the study was to evaluate how three distinct social media platforms perform regarding exposure to HPV vaccine messages. This study contributes to the vaccine communication, intervention, and implementation of science literature by describing the necessary translation steps for adapting HPV vaccine EBIs to the social media environment and gaining a better understanding of how different platforms may propagate vaccine messages.

## Methods

The EBCCP intervention was adapted and disseminated on social media over 4 months between February 14 and June 26, 2021, using three platforms: Instagram, TikTok, and Twitter. Our choice of platforms was informed by their use by the target audience of youth and young adults (1, 7) for whom HPV vaccination is recommended. Additionally, our choice of TikTok and Instagram platforms was based on these platforms disseminating primarily visual and video-based messages; hence, they align with the video format of the EBCCP.

### Video Dialogue Adaptation Process

Adaptation of the video-based EBCCP consisted of first annotating the 1- to 2-minutes video scripts according to centering theory (42), and discourse coherence theory (43); shortening the videos into 15- to 30-second segments; and adding question captions at the end of video segments to arouse curiosity and entice users to watch the next video segment. The EBCCP HPV videos consisted of dialog, typically between two young adults, but some videos also reflected dialog between a parent and young adult or a doctor and young adult. The original 1–2-minutes video dialogue, which needed to be shortened to 15–30-s videos for social media, was segmented by considering the lexical cohesion properties, topic shifts, and dialog acts used in the video scripts. Dialog cohesion is a discourse property that explains why words “stick together” in discourse (43). Also, the content of multiparty dialogs is not the only consideration, but also the form of dialog and inherent signals of topic shift (44, 45). In addition to segmenting the videos by considering the dialog between participants in the conversation, we were also interested in informing our engagement strategy by generating (a) hot keys (questions) associated with each segment and (b) have answers in the subsequent segment and, thus, imply one of the most impactful dialogs, the question and answer (46). In this way, discourse coherence and centering theory guided the video segmenting adaptation process.

### Handling of User Interaction

Although we expected comments, few authentic and organic comments were posted that went beyond emoji posts. Spam and misinformation comments were deleted. Authentic comments included positive emojis and favorable comments like “isn't science great” or “Coloradoteensforvaccines great vid!” On one occasion, two women commented that they contracted HPV despite being vaccinated. Responses to these comments opened with an empathic statement acknowledging the disappointment of receiving an HPV diagnosis. Empathic statements were followed by clarifying the CDC recommendations, which emphasize the importance of on-time vaccination by age 15 for the most optimal protection.

### NCI EBCCP HPV Vaccine Decision Narratives

Thirteen NCI EBCCP HPV vaccine video stories were adapted and implemented (video content details and links to view videos available in the Supplementary Material). The 13 videos included: (a) young adult peer HPV vaccine dialogue (e.g., college women having a boba drink after lecture and discussing why one of the women missed class to visit her “gyno” and received the recommendation to vaccinate against HPV); dialogue among two men playing basketball and discussing a visit to the doctor for genital warts; hair salon peer dialogue discussing the experience of a colposcopy procedure after a HPV diagnosis; small-town peer dialogue about getting the HPV vaccine shot as part of being a summer coach and the vaccine preventing throat cancer; Vietnamese-American peer dialogue about vaccinating both men and women to protect the health of romantic partners; peer LGBTQ+ conversation at pool about vaccinating as better protection than thinking HPV will clear up on its own); (b) doctor–young adult HPV vaccine dialogue (e.g., a young adult woman being hesitant to vaccinate because her mother would disapprove; LGBTQ+ dialogue with doctor that HPV vaccination is recommended for all regardless of sexual orientation; beginning to date someone romantically and vaccinating to protect sexual reproductive health regardless of a partner's sexual health history); (c) parent–young adult HPV vaccine dialogue (e.g., mother–daughter conversation about the mother's sister being diagnosed with late-stage cervical cancer; mother–daughter (preteen) HPV vaccine dialogue about the benefit and safety of vaccinating); and (d) a monolog about not knowing much about HPV but realizing how common it is and the benefits of vaccinating.

### Intervention Adaptation Strategies for the Social Media Message Environment

Twelve strategies were used to adapt the NCI EBCCP HPV vaccine intervention to the social media setting. The selection of adaptation strategies was informed by the vaccine social media literature (47–50), implementation and dissemination of science frameworks, such as RE-AIM (19, 21) and push-pull (5, 20), and empirically grounded experience by two co-authors who are social media micro-influencers. The 12 strategies are listed in Table 1. Next, we elaborate on each strategy.

**Table 1:** Social media engagement strategies employed across 4 months.

**Intervention adaptation and engagement strategies**
1. Use of strategic handle name (i.e., @realhotgirlshot) adapted from influencer Megan the Stallion (@realhotgirlsh*t) who has 26.4 million followers
2. Segmenting and shortening the evidence-based cancer control program (EBCCP) HPV vaccine videos into 30 second segments with captions and questions
3. Strategic use of platform features: reels, video length, stories, highlights, geotagging, polls
4. Use of hashtags and captions to widen reach i.e., number of followers (e.g., including links, facts, or questions in captions)
5. Use of a Linktree inserted into each account profile bio to encourage cross-platform engagement
6. Posting content frequently and regularly i.e., 3x weekly
7. Tag and follow influencers (e.g. @CDCgov)
8. Follow for Follow strategy with those that follow similar accounts
9. Engaging with similar accounts (e.g., @DenverTeensforVaccines)
10. Visual Aesthetics (e.g. use of Canva templates to deliver engaging and vibrant content)
11. Hot Keys & Segmentation of EBCCP videos
12. Use of timely COVID-19 vaccination as discussion point to engage users

#### Strategic Handle Name

We chose to name the accounts on Instagram, Twitter, and TikTok with the handle @realhotgirlshot. This handle references pop culture trends and is an adaptation of the Houston, Texas-based celebrity female hip-hop and rap artist Megan Thee Stallion, who has coined the term “real hot girl sh^*^t” in lyrics from one of her trending songs and who won three Grammy awards in 2021 for the best rap song, best new artist, and best rap performance (51). Her phrase “real hot girl sh^*^t” gained popularity in 2019, when audio from the song trended on TikTok (52). With more than 1.2 million videos using that audio on TikTok, we theorized that using this handle name and audio would bolster our vaccine promotion recognition and potential to go viral. Also known as a spoofing strategy, this approach was applied to capitalize on the name or audio recognition (53), which, in this case, reflects American youth culture. The approach to using our handle name aimed to contribute to increasing the following and sharing of the account and its postings. With our target audience being primarily youth aged 11–26 who are eligible for the HPV vaccine, along with parents, we used this handle to be relatable and “catchy” to our target audience.

#### Segmenting and Shortening NCI EBCCP HPV Vaccine Videos

We used videos, which had previously and significantly increased HPV vaccination in randomized controlled trials, to disseminate and broadcast culturally tailored HPV vaccine information. The videos were segmented by logical sequences and shortened to fit the requirements of each platform. We drew on the social affordance literature to understand how the informal social media dissemination environment and social affordances of the different platforms may enhance the diffusion of the evidence-based vaccine video segments and how these social affordances may amplify the communication process (54). For instance, TikTok only allows posting videos of up to 3 minutes long (26), whereas Instagram allows both photos and videos to be posted, along with stories, reels, and IGTV (23). Reels on Instagram can be a maximum of 30 seconds, whereas IGTV videos can be 15 minutes when uploaded from a mobile device and up to 60 minutes when uploaded from the web or a computer (55). For Twitter, videos, pictures, and links can all be shared, but any text-based content can include no more than 280 characters per tweet. The maximum length for videos on Twitter is 2 minutes and 20 seconds (56). Given this information, each HPV video was shortened to 1 minutes in the adaptation process and posted on all platforms.

#### Social Media Platform Features

Additional strategies, such as posting content on reels on Instagram or “for you” pages on TikTok and inserting mentions, were used to increase engagement depending on the video length and platform. We drew on the social affordance literature (54) to understand how aspects of these platform affordances may increase exposure to our vaccine messages among social media networks and exposure among anyone on social media (outside of networks). For Instagram, if videos were no longer than 30 s, they were posted to reels. This strategy was used to increase views because all public reels posted by a public account can be viewed in the reels tab on Instagram (55). This increases the potential of the video being viewed and can increase reach. Influencers use reels as an engagement strategy. Reels have played a key role in increasing organic reach for these users (57). The more views on a reel's video, the more potential for increased engagement and followers (57).

For TikTok, a similar strategy was used. Although TikTok does not have a reel's function, shorter videos are known to do better on the app and gain more popularity (58). Thus, videos were edited and segmented to be shorter without sacrificing the educational content. Additionally, TikTok's “for you” page functions similarly to the reels tab on Instagram. As Instagram allows any user to view a reel video posted by a public account, including users they do not follow, TikTok has a similar function. The “for you” page on TikTok allows users to engage with content from accounts they may not already follow and can help increase organic reach (34). For Twitter, videos can be up to 2 minutes and 20 seconds in length, links can be shared, and photos can be posted in a tweet. A user can include one to four photos in a tweet (59). Because Twitter does not have a “for you” page or reels tab, videos and photos were shared as tweets on this platform.

Other platform features were strategically used to increase engagement, including geotagging, stories, polls, and highlights. Geotagging was used to tag the broad location of the post. For this study, Orange County, California was mainly tagged, because much of our research team is from the University of California, Irvine and we wanted to reach local followers. Geotagging encourages engagement from followers in the tagged area (60). Moreover, potential followers can access the post if they click on or search that geotag (60). All content shared on Instagram as a post or reel was also shared on Instagram stories. This strategy was used to alert new and potential followers of our latest posts and encourage engagement. Geotagging and polls were used on stories to encourage engagement. Polls asking about intent to vaccinate were utilized. When engaging with similar accounts and micro-influencers, we also shared some of their content with our stories. If stories were relevant and created engagement, we saved them as highlights on our Instagram profile. Because we had a public profile, highlights could be seen by any user, regardless of whether they followed us (61). This allowed for further engagement with the story content because stories can only be viewed for 24 h, but highlights can be viewed as long as they are public (61). These strategies were used on Instagram because TikTok and Twitter do not have these functions.

#### Use of Hashtags, Captions, and Links to Widen the Reach

Captions and hashtags on each platform functioned differently and were adapted to fit the requirements of each platform. Captions on TikTok can be up to 100 characters and up to 33 hashtags, whereas captions on Instagram can be up to 2,200 characters and up to 30 hashtags (62). Tweets can be up to 280 characters with an unlimited number of hashtags (63). Using this information, captions on TikTok were shortened to include the minimum relevant information, such as “CDC states that the HPV vaccine is highly effective in preventing the targeted HPV types!”. Drawing on cognitive load theory (64–66), for Instagram as with the other platforms, captions were organized in an “eye-catching” way to not overwhelm the reader. We did so by spacing out sentences, including only relevant information and facts, using emojis, using a conversational style, asking questions, and including important links on both the captions and profile.

Hashtags were used on all platforms, with the maximum number of hashtags being used on Instagram due to the high character count for the caption. This hashtag strategy was not used for TikTok, because many influencers suggest that using too many hashtags on TikTok can backfire and attenuate the chances of increasing views (67): only one to four hashtags for TikTok videos were used. Because Twitter has also a smaller character limit for tweets, hashtags were used sparingly on this platform. Relevant hashtags that were currently trending and had a high follower and usage count were used across platforms. For example, trending COVID-19 and vaccine hashtags #COVID, #Pandemic, #ThisIsOurShot, and #IgotTheShot were included to increase engagement.

#### Driving Content Across Platforms

Another strategy used to increase engagement and reach included creating a Linktree account to drive content across platforms (68). Linktree is a social media reference landing page where a dedicated URL is provided to organize and reference all URL links in one space. Because many young adults use multiple social media platforms (69), Linktree showed promise to gain wider reach through cross-platform communication (70). Linktree uses links to each active account promoting HPV vaccination that is shared directly on the profiles of each account (71). Such a cross-platform communication strategy was theorized to encourage cross-platform engagement from both current and potential followers who may have engaged with our content.

#### Frequent Content Posting

To increase engagement and retention of followers, the content was posted weekly over 4 months, highlighting not only vaccine messages but other public health wellness and lifestyle events (about three times weekly) as well. Instagram's and Twitter's ability to post both videos and photos made it easier to create and share content. Strategies to post consistently and organize content were used. Content calendars, social media holidays and observances, and social media planning apps were utilized for this study. Social media vaccine intervention literature has also shown that posting on general prevention and lifestyle interest topics, and not only vaccine promotion, will facilitate retaining interest and engagement with an account (47, 48, 72). Planning apps, such as Planoly, were used to organize content and captions before posting (73). Content calendars were created to organize days for posting along with themes, holidays, and observances for each month. In addition, the content was created using free online graphic design apps, such as Canva, for “filler” posts to celebrate relevant holidays and observances. Filler posts function to help content creators post content more efficiently and consistently and to keep interested (74) (see Table 2 for sample weekly posts).

**Table 2:** Example content calendar of weekly social media themes and postings April 2021.

**Theme**	**Content post title**	**Caption**
**Week 2**		
About HPV vaccination (April 5)	Rural small town young women video (Segment 1)	Happy Monday! Did you know that HPV infections and cervical pre-cancers (abnormal cells on the cervix that can lead to cancer) have dropped significantly since the vaccine has been in use?! According to the CDC, among young adult women, infections with HPV types that cause most HPV cancers and genital warts have dropped 71 percent!! Talk to your doctor about how you can protect yourself from HPV-related cancers today. #HPV #HPVawareness #AskAboutHPV #CaHPVVaxWeek #CaliforniaHPVFree #HPVvaccine #ThisIsOurShot #COVID19 #Pandemic #HPVandCancer #HPVPrevention #PreventHPV #IgotTheShot #EducateToEradicate #HPVAlliance #Vaccine #Covid19 #CuckFancer #CervicalCancer #Vaccinate #VaccinatetoEradicate #Finals #Student #College #PublicHealth #Health #Nutrition #Selfcare #ZoomUniversity #Zoom
World health day #LetsTalk (April 7)	World health day #LetsTalk (Filler Post)	Happy World Health Day! As we celebrate today, we encourage you to remember that vaccines represent one of the most important global health achievements. According to the WHO, immunizations save approximately 2.5 million lives every year!! Take charge of your health. Talk to your doctor today about what vaccines you may need. Stay safe! #WorldHealthDay #HealthDay #Immunization #VaccineAwareness #Vaccination #HPV #HPVawareness #AskAboutHPV #HPVvaccine #ThisIsOurShot #COVID19 #Pandemic #HPVandCancer #HPVPrevention #PreventHPV #IgotTheShot #EducateToEradicate #HPVAlliance #Vaccine #CuckFancer #CervicalCancer #Vaccinate #VaccinatetoEradicate #Health #Nutrition #Wellness #Selfcare #Zoom #Quarantine
About HPV vaccination (April 9)	Rural small town young women video (Segment 2)	Happy Friday! Did you know that previous studies indicate the protection provided by HPV vaccine is long lasting?! According to the CDC, studies have followed people who received HPV vaccine for about 10 years, and protection has remained high in those individuals. There has been no evidence of the protection decreasing over time either! Talk to your doctor about how you can protect yourself from HPV-related cancers today. #HPV #HPVawareness #AskAboutHPV #CaHPVVaxWeek #CaliforniaHPVFree #HPVvaccine #ThisIsOurShot #COVID19 #Pandemic #HPVandCancer #HPVPrevention #PreventHPV #IgotTheShot #EducateToEradicate #HPVAlliance #Vaccine #Covid19 #CuckFancer #CervicalCancer #Vaccinate #VaccinatetoEradicate #Finals #Student #College #PublicHealth #Health #Nutrition #Selfcare #ZoomUniversity #Zoom

Organic content (content published that is not advertised or paid) from the EBCCP was disseminated regularly. The vaccine content was culturally grounded and originally drawn from interviews. The end of videos contained cues-to-act tag lines to prompt action, i.e., schedule vaccination or talk to a health care provider about vaccination and ask questions.

#### Tag and Follow Influencers

Other strategies used to increase engagement across platforms included tagging and following influencers, “follow for follow,” and engaging with similar accounts and influencers. Following influencers in the public health field, such as @CDCgov or celebrity rapper Megan Thee Stallion (@theestallion), according to social affordance theory, is likely to prove helpful in building a following, network, and reach. Following and tagging accounts that are popular in a niche (e.g., @CDCgov for public health) or social media influencers, and celebrities like Megan Thee Stallion can increase the likelihood that people who do not already follow you will see your content. This strategy was used on Instagram. Tagging accounts on an Instagram post ensures content will be shown on the “tagged” tab of that account's profile (if the user allows tags to be public). This increases the potential of the post reaching the target audience.

#### Follow-for-Follow Strategy as Instagram's Unique Strategy

The follow-for-follow and following similar accounts strategies were used on all platforms. This strategy included following every account that followed us to build engagement, trust, and retention of followers. Initially, as a new account, we needed to build a following from scratch. To do so, we chose to follow individuals who followed similar accounts. If individuals were interested in similar topics, they were more likely to engage with and follow our account.

#### Engage With Similar Accounts

Following similar accounts and influencers regarding vaccines, COVID-19, health, and HPV and tagging those accounts helped increase potential content views and engagement by building partnerships through a follow-for-follow strategy. By following similar accounts (e.g., @DenverTeensForVaccines) and engaging with their content, mutual trust was formed to help build engagement. These similar accounts essentially engaged with our content if we did the same. Overall, the following accounts that had similar content regarding providing information on vaccine hesitancy or HPV were useful for increasing reach. The more likely other accounts engage with your content, the more likely your content reaches your audience.

#### Visual Aesthetics and Branded Logo

**Figure 1:**
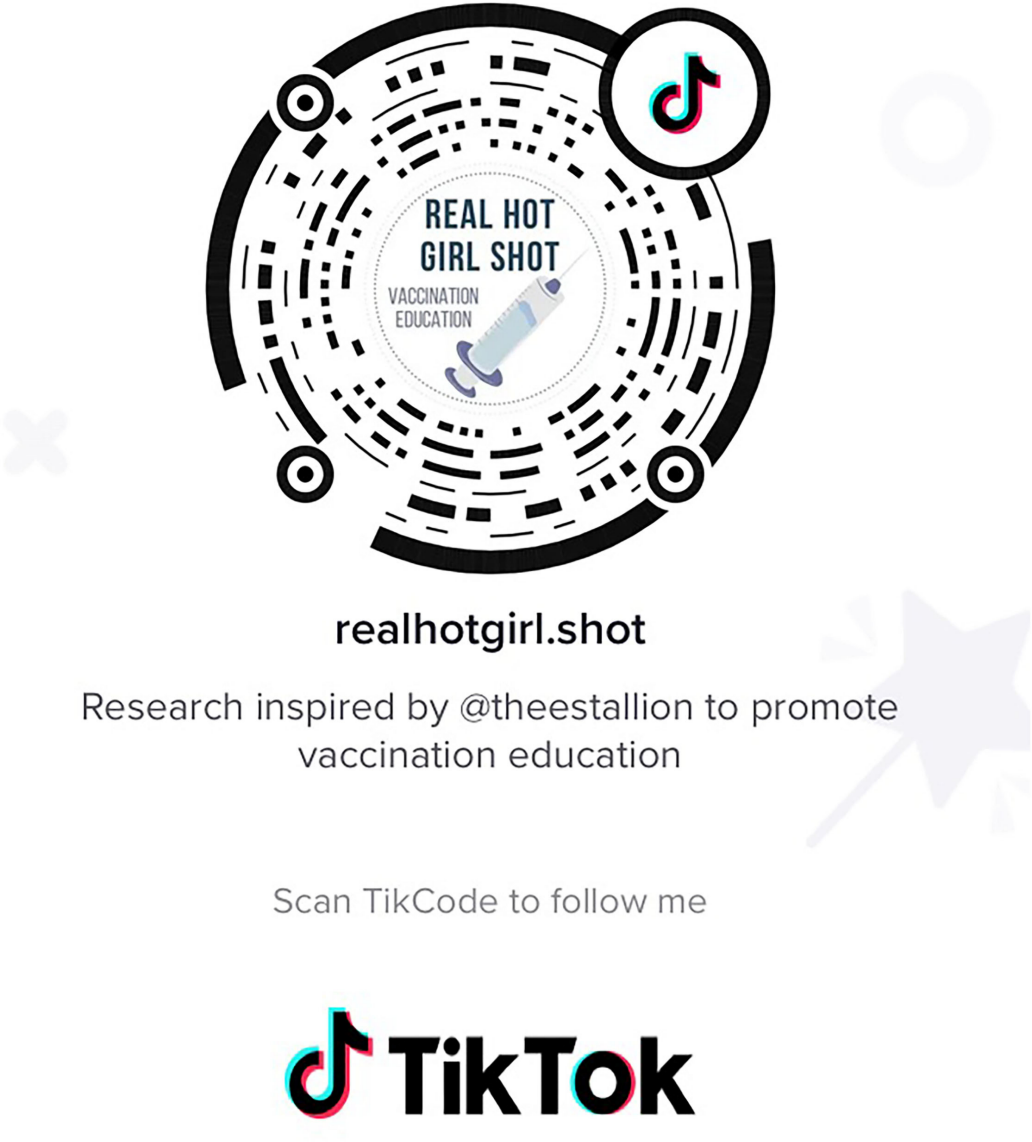
Branded logo for social media.

Visual aesthetics are important in creating a “branded” account that users will trust and want to follow. We created a QR code logo (see Figure 1) that was shared as the profile picture on all platforms to create a uniform brand identity. The username @realhotgirlshot was used as “Real Hot Girl Shot” with the title “Vaccination Education.” The business account was categorized as an educational platform on Instagram and TikTok. Using Canva, covers for the highlights were created using similar branding and fonts to the logo we created. Canva was also utilized to create filler posts and other aesthetic content. Its wide range of free templates made creating vibrant and engaging content much easier.

#### Hot Keys and Segmentation of the EBCCP HPV Vaccine Videos

**Figure 2:**
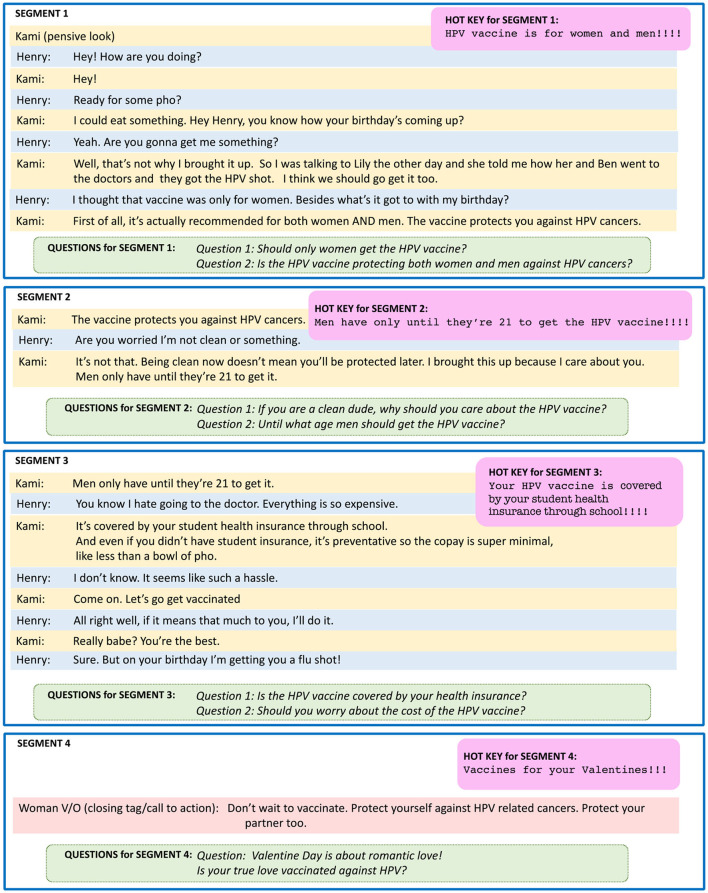
Example of the segmentation of an EBCCP HPV video, showcasing the hot keys and questions associated with each segment.

Centering theory (42) assumes that each discourse segment evokes some entities, ideas, or other abstractions, referred to as centers. The theory provides a mechanism for scoring these centers, such that the highest scoring center becomes the “preferred center.” Each discourse segment has a different preferred center. We hypothesized that the preferred center of each segment of an EBCCP HPV video can be used to generate a hot key for each segment. As shown in the example illustrated in Figure 2, the first segment concerned three centers in this example of HPV vaccine video set in Little Saigon, a Vietnamese District in Southern California: *C*_1_ = the gift for Henry's birthday, *C*_2_ = the HPV vaccine, and *C*_3_ = a bowl of pho. As implied by the character Kami but not directly expressed, she intended to offer *C*_2_ as *C*_1_, hence *C*_2_ becomes the preferred center for Segment 1 of the EBCCP HPV video, illustrated in Figure 2. In addition, the utterances of Segment 1 offered an important theme of *C*_2_; that it is recommended for both women and men. This theme of *C*_2_informed the hot key that we created for Segment 1. This explains why the two questions generated for Segment 1 (using them as captions) referred to the beneficiaries of the HPV vaccine. Both were yes-no questions, but they also raised awareness of the confidence in the vaccine, manifested by Kami and vaccine literacy, which Henry lacked because he was unaware that vaccinating was also recommended for men.

Note that Video Segment 2 started with the same last utterance Kami had in Segment 1. In this way, the two-party dialog remained coherent. Moreover, center *C*_2_ looked forward to Segment 2, where a new center was introduced: *C*_4_ = Henry being “clean” (in relation to sexual health). However, a causal coherence relation was established between *C*_2_ and *C*_4_, ensuring that *C*_2_ (the HPV vaccine) remained the preferred center even in Segment 2, but with a different theme, namely, one that temporally constrained it, requiring vaccination for men to occur before they turn 21.

As in Segment 1, the hot key for Segment 2 was informed by the new theme of temporally constraining the HPV vaccine and, thus, addressing the factor of vaccine complacency. However, this time, the questions that were generated were no longer yes-no questions. In fact, Question 1 addressed the causal explanation between *C*_2_ and *C*_4_, engaging participants to elaborate on their understanding of HPV vaccine complacency and manifest their vaccine literacy. Question 2 pinpointed the temporal constraint introduced as a new theme for the center of *C*_2_: the HPV vaccine.

Segment 3 considered the affordability of the HPV vaccine, yet another theme informed the hot key. It also introduced two new centers: *C*_5_ = vaccination cost and *C*_6_ = health insurance. Interestingly, when *C*_5_ was introduced in Henry's utterance, it showed how this explains his hesitancy. Viewers of this EBCCP HPV video segment might identify with Henry. Therefore, it is important that when *C*_6_ was introduced immediately after Kami, it offered a solution to Henry's hesitancy. When Henry continued being on the fence about vaccination, he referred to *C*_2_. Through the pronoun “it,” the segment maintained that this center was the focus of attention. When Kami referred in the next utterance to vaccination, by giving a cue to action, *C*_2_ became the preferred center, yet in relation to *C*_6_, which informed the hot key of segment 3. Question 1 associated with this segment was a yes-no question, reinforcing the relation between *C*_2_ and *C*_6_, addressing vaccine affordability. Question 2 further connected vaccine affordability to vaccine complacency by using the relation between *C*_2_ and *C*_5_.

Segment 4 consisted of a single utterance, provided by another voice, building confidence in the HPV vaccine and its effectiveness in protecting against HPV cancer. This time, the hot key was generated differently. Because *C*_1_ (the gift for Henry's birthday) was the first center introduced in the dialog, referred to again in Segment 3 when Henry proposed a flu shot as a gift for Kami, we chose the hot key to be a gift for a special occasion. Because we were launching this segment before Valentine's Day, we selected it as the special occasion. This idea was reiterated in the question associated with this segment: “Valentine's Day is about romantic love! Did your partner get their HPV vaccine yet?”.

#### Use of COVID-19 Vaccination as a Conversational Entry Point

In addition to the strategies discussed, vaccination for COVID-19 was used as a conversational entry point to guide our audience toward HPV vaccination messages. For instance, dialog on all platforms, including captions, story posts, and tweets were framed around discussions regarding COVID-19 vaccination. Posts focused on the pandemic anniversary in March 2021 emphasized the importance of getting vaccinated, following CDC guidelines, and tackling misinformation. Debunking COVID-19 myths were shared. The topic of COVID-19 vaccination was used as a conversational segue to talk about HPV vaccination. For example, a 71% decrease in health care visits for vaccination including HPV occurred during the COVID-19 pandemic (75). Content that was posted regarding low vaccination during the pandemic emphasized the importance of prioritizing HPV and COVID-19 vaccination. Pandemic hashtags were used on all posts to emphasize this point, including #IGotTheShot, #ThisIsOurShot, #COVID19, and #Pandemic.

### Data Analysis for Evaluation of Intervention Dissemination

Data for the evaluation of the observational study were retrieved from each respective platform's application programming interface and in the case of Instagram and TikTok, from business accounts with these two platforms. The business accounts made aggregate-level data accessible on engagement, the number of followers, reach, and impression metrics. Longitudinal data on the same individual users was not available. The descriptive and inferential statistics were performed to examine whether there were significant differences in the strategies used. Frequencies on mean accounts reached, and independent sample *t*-tests were performed accounting for unequal variances (Welch's *t*-test).

## Results

### Platform Performance

Instagram outperformed TikTok and Twitter during the 4-month observational study period in Spring 2021 by receiving the greatest number of impressions, followers, and engagement, whereas TikTok reached the greatest number of unique accounts who viewed the handles' HPV vaccine messages (see Table 3).

**Table 3:** Social media platform performance.

	**Impressions (# of times content was seen, viewed, or played on the app)**	**Reach** **(# of unique accounts that have seen our content)**	**Followers**	**Engagement** **(likes, shares, retweets)**
Tik Tok	8,327	8,614	184	441
Instagram	9,986	7,998	389	621
Twitter	5,659		17	163

### Evidence for Intervention Adaptation and Dissemination Strategies That Increase HPV Vaccine Message Exposure

For Instagram, the top performer on engagement, impressions, and followers, evidence supporting the use of 6 of the 12 dissemination strategies was found for increasing the number of accounts reached. In reference to Table 1 listing of dissemination strategies, we found evidence for #3 strategic use of platform features, #4 use of hashtags, #6 posting content frequently, #7 tag and follow influencers, #11 hot key segmentation of EBCCP videos, and #12 use of timely COVID-19 vaccination as a discussion point to engage users. We elaborate on the evidence for these strategies next.

#### Strategic Use of Platform Features

**Figure 3:**
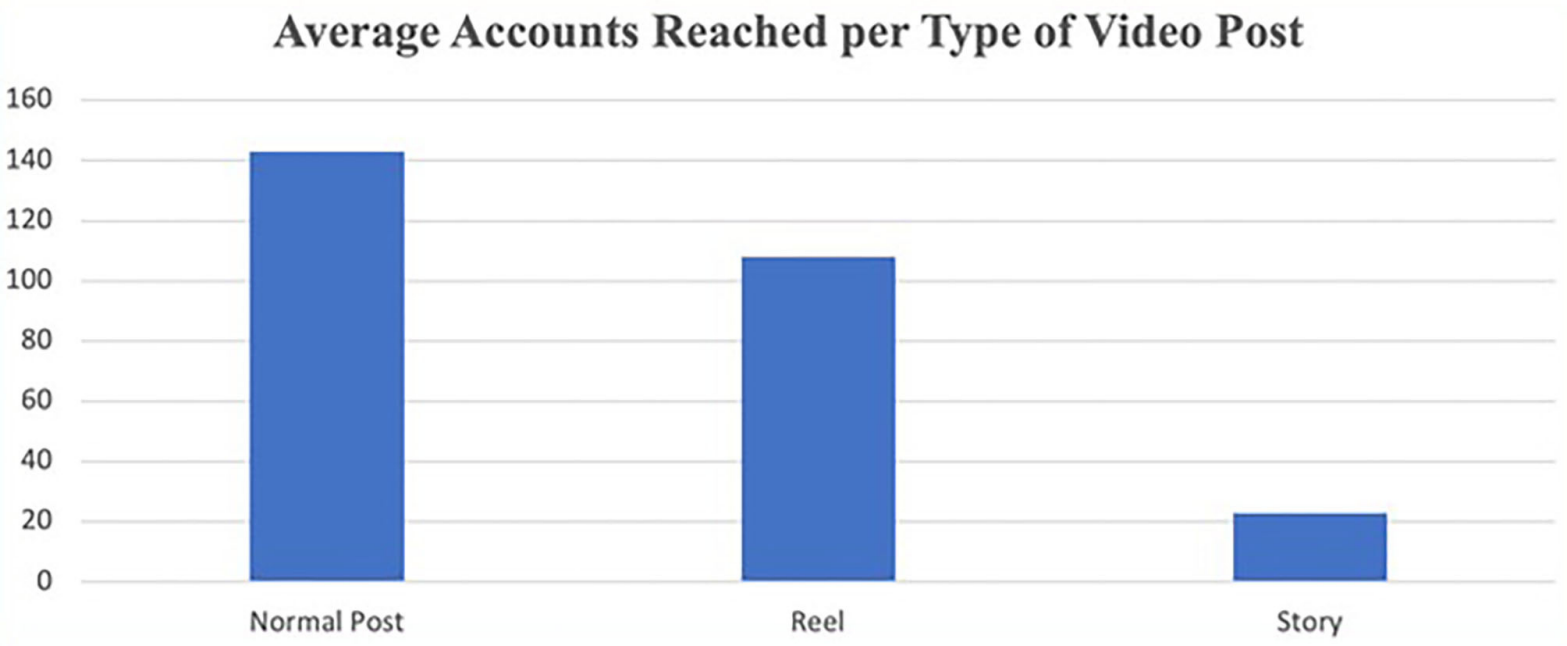
Average accounts reached per video post type.

Examining platform features on Instagram showed that although posting videos as reels (where anyone can view the video, not only followers of the account) or stories increased reach, normal posts reached a significantly greater number of accounts on average (143 ± 40 normal accounts reached) compared to posting content on reels (108 ± 54 accounts reached), or as a story (23 ± 9 accounts reached) (see Figure 3). The relationship was significant when comparing normal with story posts (*t* = 20, *df* = 47, *p* < 0.001), but was not significant when comparing normal posts with reels or story posts (*t* = 1.7, *df* = 4, *p* = 0.157). Normal posts reached 6,583 accounts, whereas story posts reached 1,729, and reel posts reached 541 accounts.

#### Use of Hashtags

The use of hashtags significantly contributed to the dissemination of HPV vaccine messages. We found that for Instagram, posts using more than 11 vaccine-related hashtags (11.7) reached a greater number of accounts on average (140 ± 42) compared to posts using fewer than 11 hashtags (23 ± 9). The hashtag strategy was significant for increasing the average number of accounts reached (*t* = 19.6, *df* = 53, *p* < 0.001). The most accounts reached occurred in the fourth month of the observational study on a post about international women's day, reaching 255 accounts indicating an upward trend as the vaccine educational account gradually began building a following.

#### Posting Content Frequently

**Figure 4:**
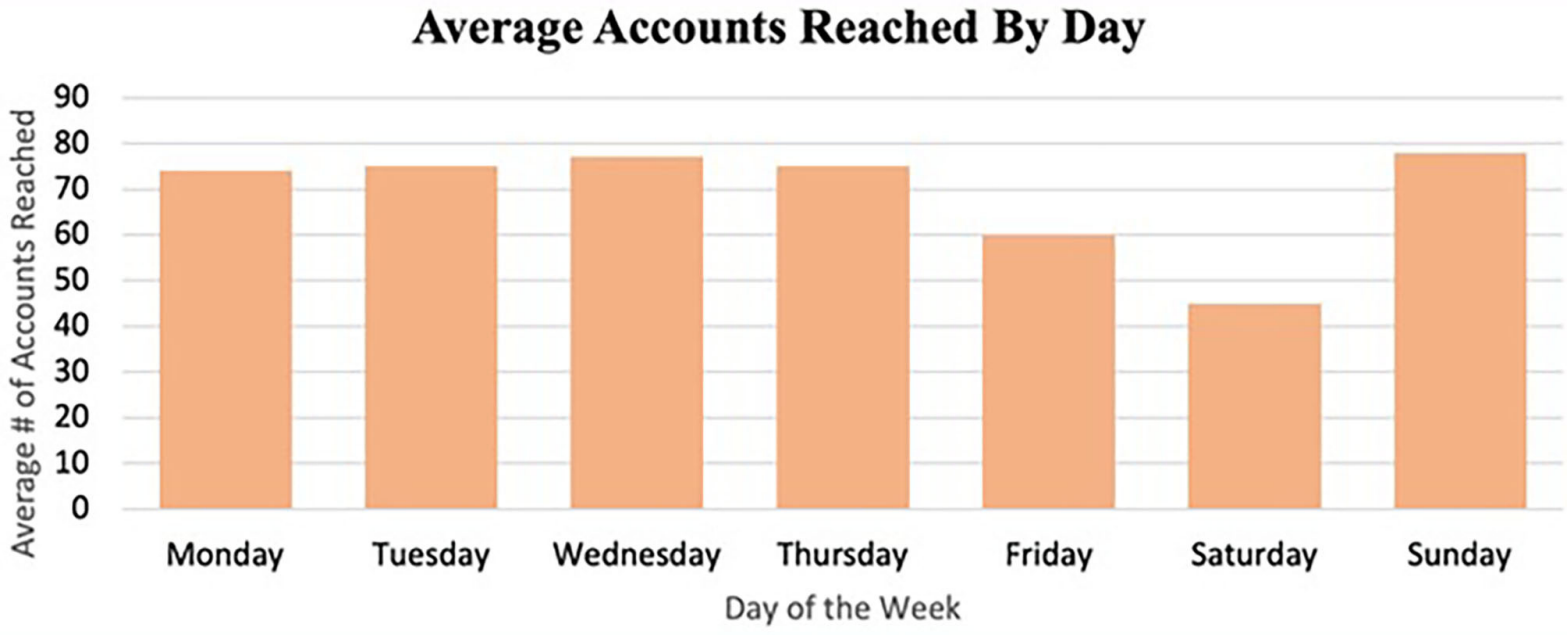
Average accounts reached by day.

**Figure 5:**
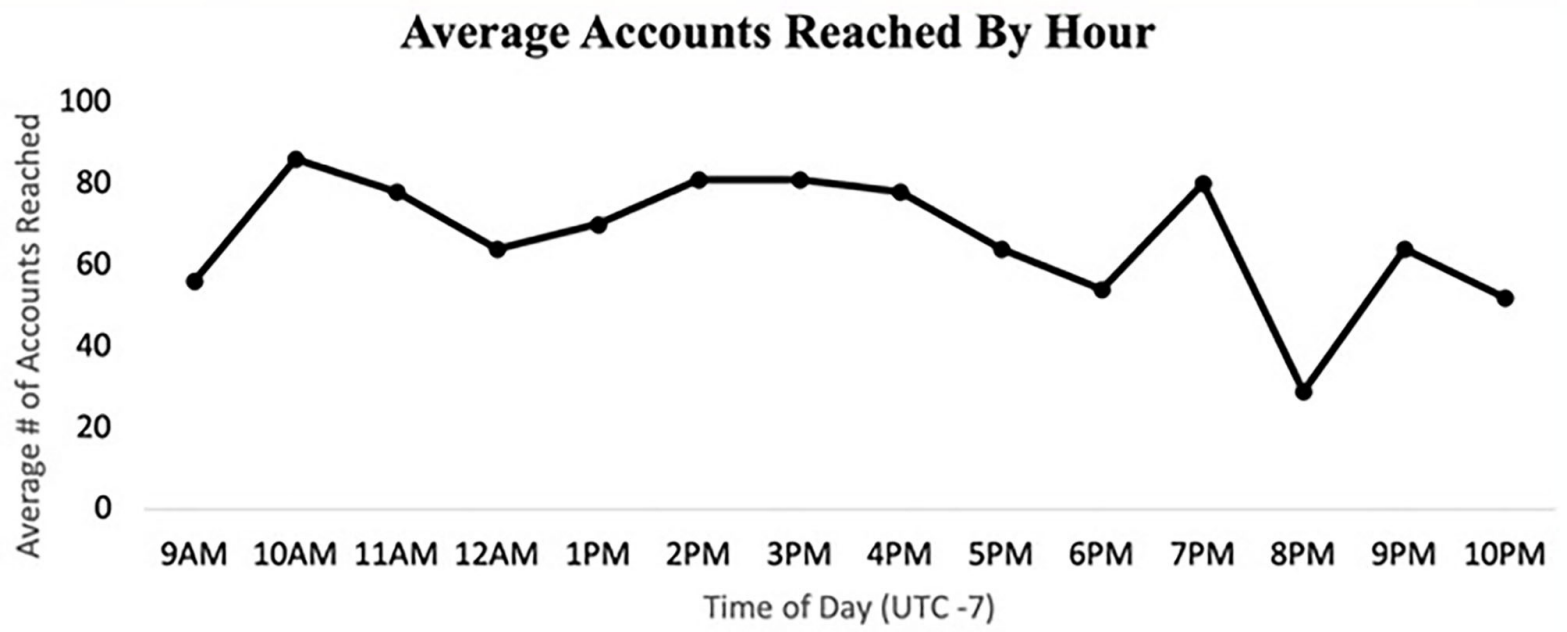
Average accounts reached by an hour.

Posting content frequently, another dissemination strategy, is a necessary strategy in the competitive social media message environment. Additionally, the timing of posting matters on both days of the week and time of day. Posting content Sundays through Thursdays reached a greater number of average accounts. As for the time of day, posting mornings around 10 a.m., afternoons between 2 and 4 p.m., and evenings around 7 p.m. reached a greater number of accounts (see Figures 4, 5).

#### Tag and Follower Influencers

For tag-and-follow influencer strategies, posts that included mentions of other accounts reached a significantly greater number of accounts on average (142 ± 26 accounts) compared with posts that did not include mentions (69 ± 64 accounts; *t* = 5.3, *df* = 40, *p* < 0.001). Engaging with similar accounts resulted in a modest 309 accounts following our “realhotgirlshot” account. Following individuals who followed HPV vaccination accounts (145) resulted in the greatest increase in followers. This was the most successful strategy in securing an audience in a short amount of time and was the source of the greatest proportion of followers. Followers included accounts representing educational groups, community organizations, nonprofits, podcasts, and informational accounts.

#### Hot Key Segmentation of EBCCP Videos

**Figure 6:**
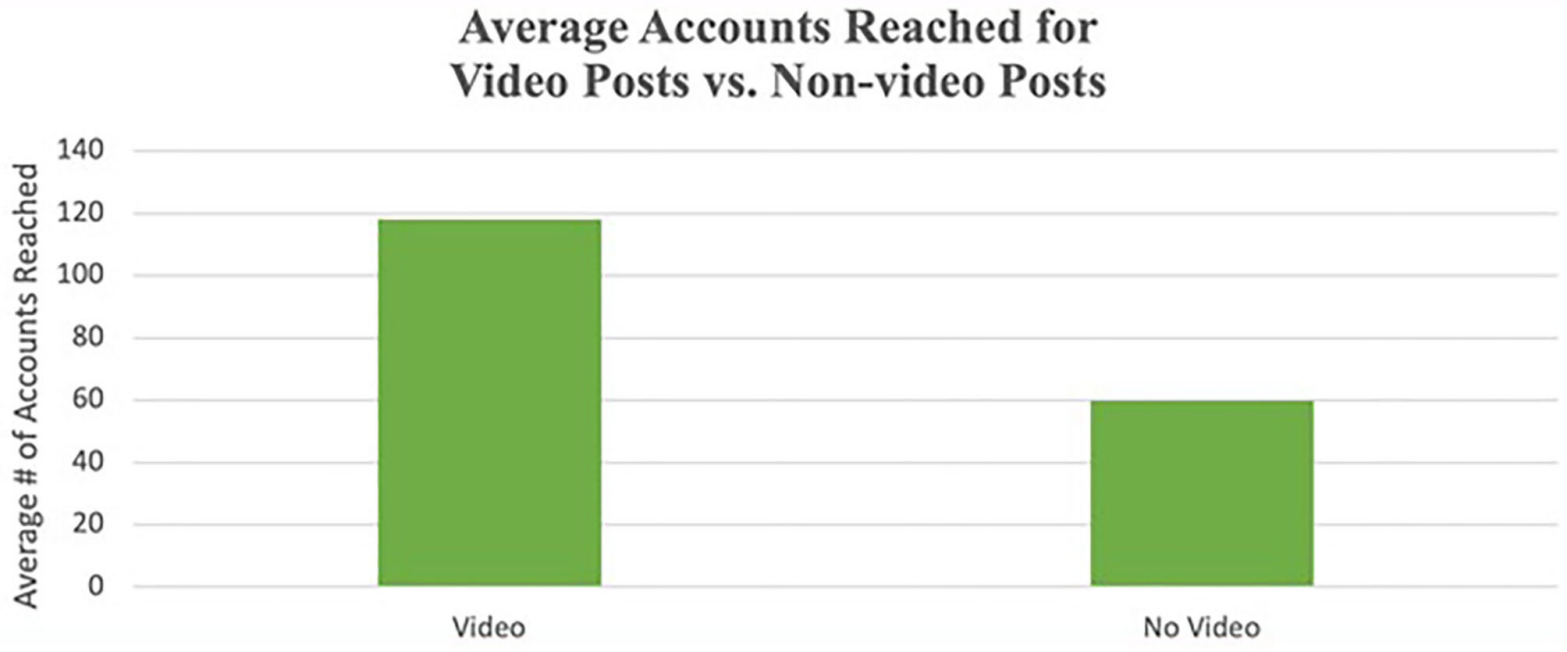
Average accounts reached for video posts vs. nonvideo posts.

Support for the dissemination of the segmented (shortened yet sequentially delivered) HPV vaccine story videos reached significantly more accounts (118 ± 42 accounts) compared to posts with no video (60 ± 63 accounts; *t* = 5.26, *df* = 40, *p* < 0.001; see Figure 6). Posting videos on the Instagram reels option and the in-feed grid led to higher views or plays compared to posting videos to reels only or in-feed grid only (see Supplementary Material that shows views by video segment). The breakdown of HPV video segments comparing Instagram and TikTok showed that the video segments consistently received more views on the TikTok platform compared with Instagram (see Supplementary Material, for details on views per video segment). Two videos, the boba HPV vaccine discussion and the clinic Latina stories, received considerably more views (2,627 views for boba and 1,169 views for clinic Latina) on TikTok than on Instagram (see Supplementary Material).

#### COVID-19 Vaccination as Discussion Engagement

Using COVID-19 posts and hashtags to draw attention to the vaccine account significantly increased the accounts reached (149 ± 1) compared to vaccine posts that did not mention COVID-19 (68 ± 64; *t* = 14, *df* = 125, *p* < 0.001). Additionally, use of COVID-19 hashtags resulted in reaching significantly more accounts (140 ± 42) compared with non-COVID-19 posts (23 ± 9; *t* = 19.6, *df* = 53, *p* < 0.001).

## Discussion

This observational study generated some of the first evidence to our knowledge, on which intervention dissemination strategies can increase user engagement and reach for educational HPV vaccine messages in a real-time, *in vivo*, competitive social media message environment. Although more difficult to measure, learning how promotional and narrative or dialogue-based vaccine messages perform in the competitive social media message environment lends greater external validity (21) and advances science about which strategies increase reach and engagement under conditions of message competition. This study generated evidence for how select social media platforms function to propagate vaccine messages. Six of 12 dissemination strategies were found to increase reach, engagement, impressions, and followers on Instagram, a platform well-suited to deliver vaccine video stories given its emphasis on visual delivery and primary use by young adults and parents—the target audience for HPV vaccine messages. Findings demonstrate that the inclusion of narrative EBCCP HPV vaccine videos contributed to significantly increasing the average number of accounts reached. Centering theory informed the strategy to coherently segment the original HPV vaccine videos into shorter videos that could be delivered sequentially.

### Lessons Learned

#### Evidence for Strategies That Increase Reach and Followers

The study generated evidence for strategies that increased the number of followers, impressions, and engagement on Instagram, whereas TikTok's video-exclusive platform increased reach (views among unique accounts) to a greater extent across all video segments during the 4 months. For Instagram, several strategies broadened the reach and increased accessibility of the HPV vaccine messages: segmenting videos and emphasizing specific vaccine messaging with captions or questions, using more hashtags including COVID-19 hashtags, posting normal posts frequently during certain times of the day and on certain days of the week, and using tag-and-follow influencer strategies (e.g., mentioning and following individuals and groups that follow HPV vaccine accounts, including educational groups, community organizations, nonprofits, podcasts, and informational accounts). This observational study, therefore, generated evidence for how the social affordances of these three platforms may increase access and reach of HPV vaccine messaging (54, 76).

#### Engagement Presents Greater Challenges on Visual and Hedonistic Platforms

Engagement, by contrast, occurred during the 4-month observational study, but it was muted in its expression by users on all three platforms. The two visually dominant platforms, TikTok and Instagram, have a greater click culture by reacting with emojis, but less so by reacting with extensive linguistic expressions (70). According to the push-pull communication framework, HPV vaccine messaging was predominantly broadcast, with little organic pull dialogue occurring on these platforms. Organic, user-generated pull communication explained by social affordance theory was not observed. Whether this is because of the topic or other explanations is unknown. Our study's low engagement stands in contrast to a 10-week statewide (South Carolina) social media HPV vaccine campaign on Twitter and Facebook that experienced pro- and anti-vaccine comments to a greater extent (48). However, it is noteworthy that both this campaign and a yearlong Danish social media campaign used paid content to push out content (47). Future research will need to explore what strategies can increase engagement. Network influencers and entertainment memes may be additional key strategies for deepening interest for greater engagement (5, 77, 78). Results brought attention to how dissemination strategies play out differently depending on the platform and the likely need for monetary incentives to respond with questions if unvaccinated. The high-quality HPV vaccine videos were intended to evoke interest and curiosity. For four months, the Instagram posts generated nearly a million impressions (the number of times vaccine messages were seen), reached nearly 8,000 unique accounts, and built a modest following of 389 accounts.

#### Tag-and-Follow Influencer Strategies and Engaging With Similar Accounts

Although we did not reach mega-influencer Megan Thee Stallion for endorsement of our vaccine account, following local micro-influencers in Orange County, California and public health and tagging HPV vaccine accounts boosted our following and reach. Social media influencers are known to improve marketing for many brands (79, 80). Influencers may be micro- (1,000–10,000 followers), macro- (10,000–1 million followers), or mega- or celebrity influencers (more than 1 million followers) (81), have differential influence, and may only endorse vaccine messages if they receive monetary compensation. With an account focused on vaccination, education, and public health, working with influencers who are social activists and care about similar topics may offer one strategy to increase following and engagement with the account.

#### Cross-Platform Behavior Is a Modest Strategy to Increase the Reach

Cross-platform user engagement was a strategy we expected to increase our following. Although having a Linktree in the bios did drive modest cross-platform activity (124 views), TikTok has a built-in option to cross-link with Instagram, while Twitter has hyperlink options to drive cross-platform messages. TikTok and Instagram do not permit hyperlinks. Overall, the Linktree cross-platform engagement strategy did not significantly increase following or reach but could still be considered to encourage cross-platform traffic.

#### Planning and Time for Building a Following and Influence

Real-time, *in vivo* social media interventions (i.e., in the dynamic message environment as opposed to implementing an intervention in a controlled social media environment) are both tricky and interesting, and many lessons were learned from the 4-month observational study. Planning for the time it takes to develop a following, networks, influencers, posting strategies, and content must all be considered in the planning of social media interventions, especially given the new communication paradigm of messages propagating primarily through peer networks (8, 10, 81). Just as it takes time to build real-world communities, the same is true for online ones (48, 82). Developing a following can take many months with routine posting and can present challenges, particularly for an account with no name recognition, influencers promoting the account, or viral content. This has also been the case with the few HPV vaccine observational social media studies (47, 48). The adaptation strategies we implemented offer an approach to begin establishing a community, but the time and effort it takes to become an influencer can present challenges. Engaging with similar accounts, establishing relationships with other users, following similar accounts, and creating content seem necessary ingredients. Because social media apps do not rest and new content is continually pushed out by other accounts, posting content continuously and engaging with others online is necessary to establish a strong following and network. Therefore, researchers conducting similar research should keep in mind that it can take several team members to manage a single account and several months to establish even a modest following. Having adequate time, planning, and resources to manage the account and engage with others is paramount for social media.

#### Posting on Specific Days and Times

Posting on specific days and times that followers are on the app is an important strategy that we could not take advantage of fully. As it takes time and effort to manage the accounts and posting on specific days and times can be difficult. The days and times when many followers are on the app are viewable in the analytics for TikTok and Instagram. However, these days and times vary between platforms, and researchers were not always available to post during that time. Online applications exist that can automatically post preplanned content and captions on Instagram for both in-feed posts and stories (83).

#### Repostable Content

Creating “repostable” content is also an important strategy. Content that is reposted means it is shared more often and more likely to be seen and, thus, more likely to go viral. The type of content posted, how relatable it is, and how relevant it is to pop culture is something to consider because contents, such as memes and reels, are known to garner the most exposure on Instagram and Twitter (77, 84). In the future, we aim to create more relatable yet educational content that is likely to be shared and reposted by other users on these platforms. This will likely increase engagement and reach (47, 48, 85).

#### Sustained Engagement and Implications for Adapting Public Health Interventions

Another unknown is the different rates of growth and engagement on each platform. In this case, Instagram had the highest rate of growth and the most followers and engagement through likes, comments, and shares. TikTok, on the other hand, received more views consistently across video segments and the most views for a single video, the Boba talk, and clinic Latina. By contrast, the intervention implemented on Twitter observed limited growth and the research team struggled to increase followers and engagement on this platform but was also less experienced with this platform. A social media background with technical knowledge of marketing strategies for propagating messages and effectively growing accounts is essential. Critical for sustained engagement seems to be the inclusion and posting of trends and topics other than vaccination to maintain interest. This was found to be the case in another social media study (72), where keeping the audience's interest by taking an integrative holistic approach worked to sustain engagement.

The COVID-19 vaccine posts and hashtags helped broaden the reach of our audience and make our content easier to find for those who did not follow our account but may be interested in keeping up with public health. For instance, users that followed COVID-19 or pandemic hashtags may have easier access to our content. In all, this strategy was used to capitalize on discussions regarding health and wellness, vaccinations, public health, and COVID-19. Framing discussions around topics that are already being discussed both online and offline was a simple way to pull our audience in and garner attention and engagement.

### Limitations

The research team faced challenges in the design, implementation, and evaluation of the study. In particular, growth and going viral on Instagram, Twitter, and TikTok can be time-consuming and requires unique strategies that dynamically respond to upcoming current events. It was difficult to solicit engagement through likes, comments, and shares from our target audience, especially because of the public nature of the accounts and low handle recognition. In addition, with only aggregate data available, it was not possible to measure whether individual users watched consecutive video segments.

The dosage of intervention exposure could not be measured directly, nor could we assess whether the same individuals followed the sequential posting of segmented videos. Future research will feature a randomized trial design and examine dose-response effects. Platform choice may need to be guided by those that provide metrics with a more granular analysis of individual users. Because of the public nature of the accounts, it was impossible to measure how much exposure each user had to the educational content on Instagram, Twitter, and TikTok.

## Conclusion

Social media offers a communication tool for disseminating and interacting with youth and young adults about HPV vaccination, given their daily message exposure on these platforms (85–87). Preventing cancers caused by HPV through vaccination remains a significant public health priority in the United States (16). Given that young adults' daily exposure to social media typically encompasses lifestyle, identity, and entertainment messages, vaccine intervention strategies that evoke interest and curiosity about HPV vaccination in the competitive message environment will be needed (87–90). Social media is a powerful tool that has the potential to revolutionize health interventions if used correctly (77, 78, 87, 91, 92), with social media being dubbed the new vital sign (93). Dissemination of HPV vaccine messages will differ depending on the social media platform, but different strategies and a comprehensive strategy are recommended to adapt vaccine interventions, build a following, and increase reach to connect with potential users who may benefit from HPV vaccine messages.

## Data Availability Statement

The raw data supporting the conclusions of this article will be made available by the authors, without undue reservation.

## Author Contributions

SH contributed to the study design conception and theory, the development of the adapted intervention, data analysis, and drafting of the manuscript. KP contributed to the social media planning, creation, and dissemination of weekly content posting, data retrieval and analysis, and drafting of the manuscript. MW contributed to data retrieval and analysis. HV contributed to the creation of and weekly posting of social media content and drafting of the manuscript. SA contributed to making graphs and figures and some content posting. SMH contributed to the development of original concepts and theory informing intervention adaptation strategies for the social media study and drafting sections of the manuscript. All authors contributed to the article and approved the submitted version.

## Conflict of Interest

The authors declare that the research was conducted in the absence of any commercial or financial relationships that could be construed as a potential conflict of interest.

## Publisher's Note

All claims expressed in this article are solely those of the authors and do not necessarily represent those of their affiliated organizations, or those of the publisher, the editors and the reviewers. Any product that may be evaluated in this article, or claim that may be made by its manufacturer, is not guaranteed or endorsed by the publisher.
