# Supplementary material for: Adaptation and Dissemination of a National Cancer Institute HPV Vaccine Evidence-Based Cancer Control Program to the Social Media Messaging Environment
Source: Front Digit Health. 2022 Jul 27;4:819228. doi: 10.3389/fdgth.2022.819228 (PMC9363572; doi:10.3389/fdgth.2022.819228)
Supplement: Supplementary file 1 [file Data_Sheet_1.docx]

Supplementary Material

# Supplementary Data

Description and links provided of the National Cancer Institute (NCI) HPV vaccine narrative video-based intervention, which has been recognized as an evidence-based cancer control program (EBCCP). Thirteen videos used for the social media adaptation study are provided: Their titles, brief description and a link to the original 1-2 minute videos.

1. Boba & HPV vaccine conversation among 2 college women

- <https://vimeo.com/313420587/c5a0ad5d89>

1. Basketball conversation among two young adult men

- <https://vimeo.com/312449526>

1. Young adult woman and doctor – started seeing someone romantically

- <https://vimeo.com/317621503>

1. Monologue – Not knowing what HPV is and what the vaccine protects against

- <https://vimeo.com/317685534>

1. Clinic Latina – young adult Mexican-American woman and OB/GYN doctor, mom’s approval

- <https://vimeo.com/313469138>

1. Mother-daughter kitchen conversation – cancer story

- <https://vimeo.com/312458191>

1. LGBTQ+ young adult woman doctor conversation

- <https://vimeo.com/313426880>

1. LGBTQ+ Pool talk about HPV vaccination

- <https://vimeo.com/312431638>

1. Peer talk kitchen conversation about colposcopy

- <https://vimeo.com/317677313>

1. Mother-daughter (preteen) talk about HPV vaccination (baking cookies)

- <https://vimeo.com/311833674>

1. Little Saigon peer conversation about importance of getting HPV vaccine even for men

- <https://vimeo.com/310818805>

1. Rural, small town peer dialogue about HPV vaccination

- <https://vimeo.com/313321535>

1. Hair salon conversation about HPV vaccination and HPV risk for African American women

- <https://vimeo.com/312639452>
