# Supplementary material for: Adaptation and Dissemination of a National Cancer Institute HPV Vaccine Evidence-Based Cancer Control Program to the Social Media Messaging Environment
Source: Front Digit Health. 2022 Jul 27;4:819228. doi: 10.3389/fdgth.2022.819228 (PMC9363572; doi:10.3389/fdgth.2022.819228)

# Supplementary Instagram and Tik Tok Engagement Tables for Dissemination of HPV Vaccine Intervention Videos


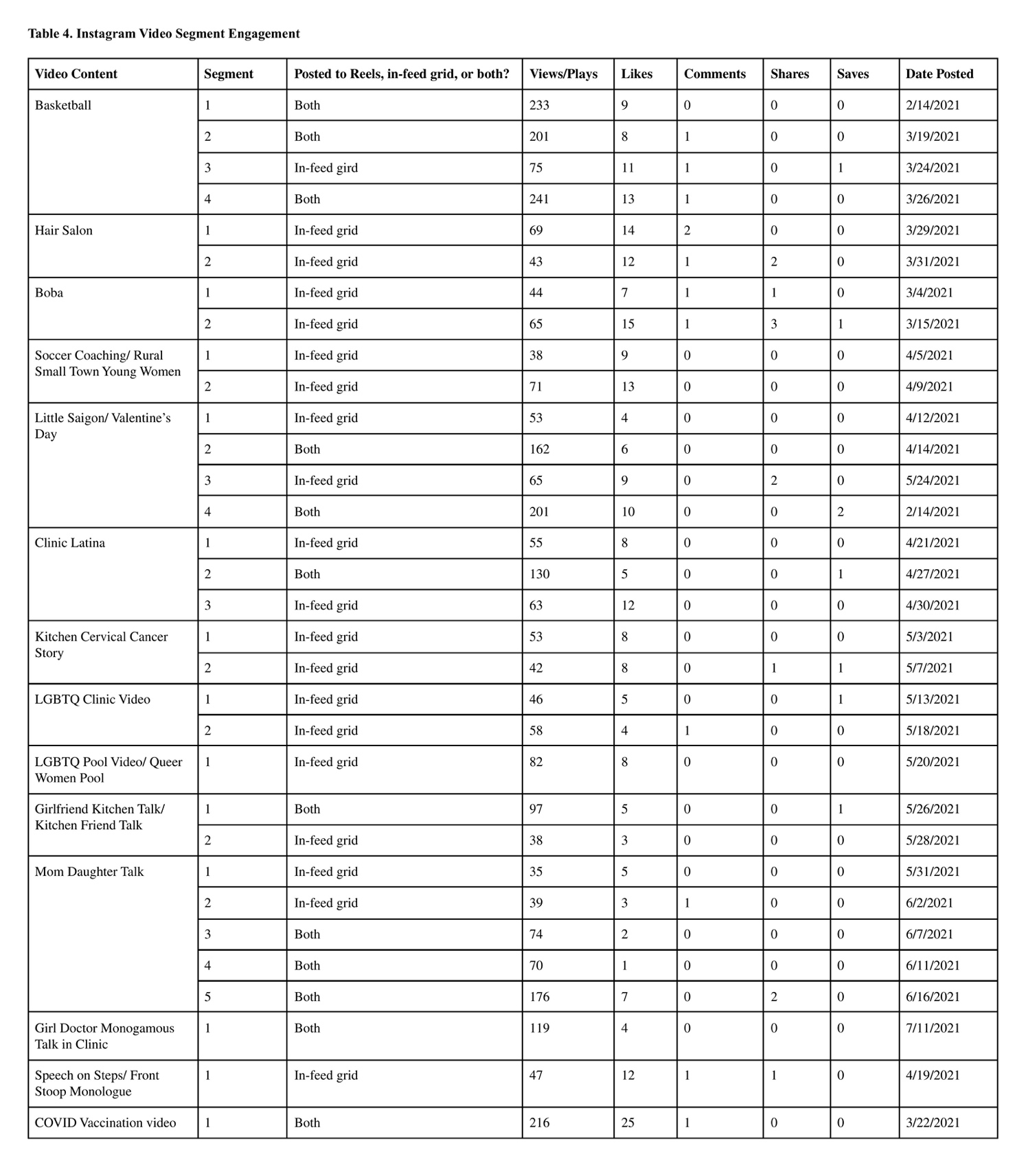


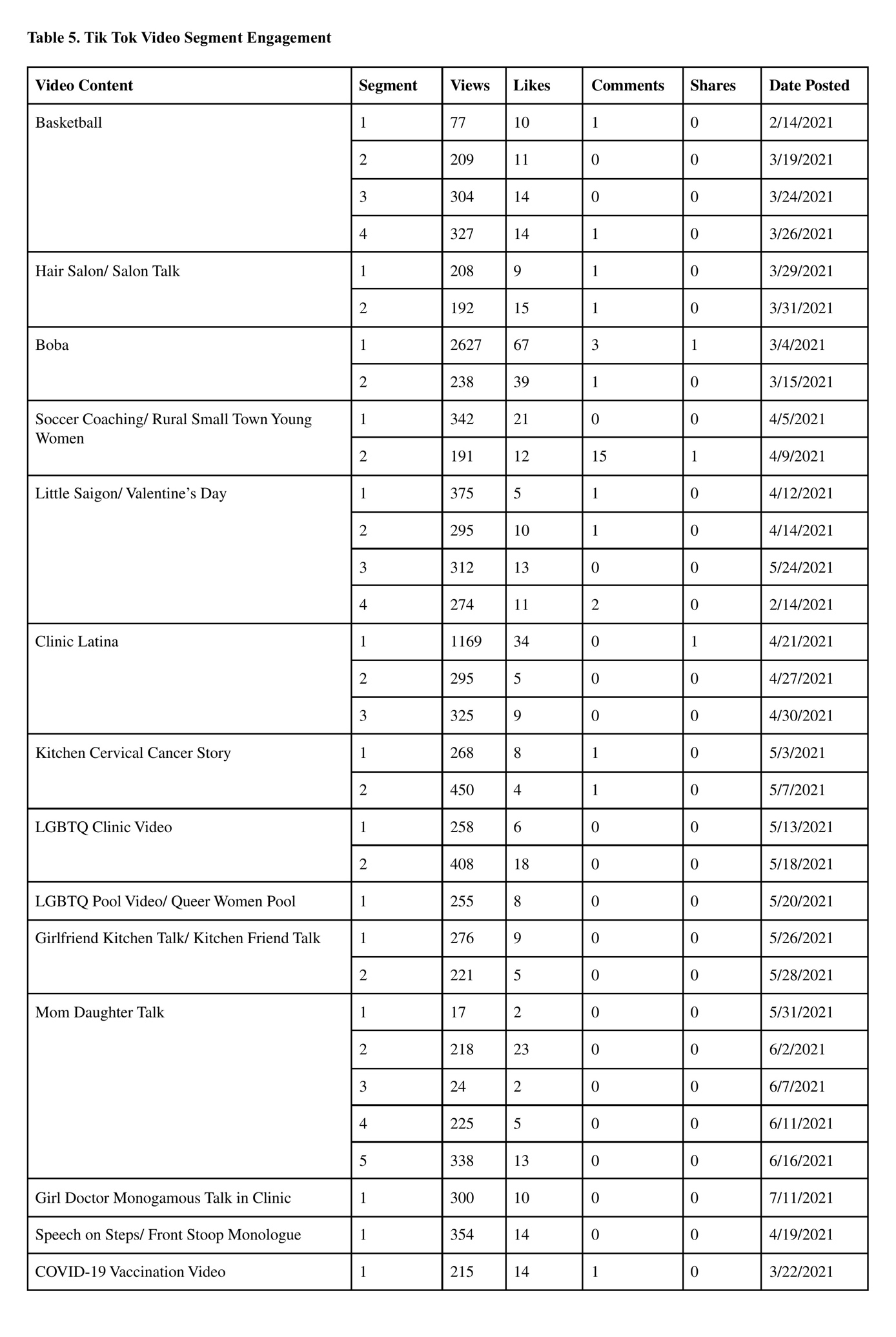

Supplement: Supplementary file 2 [file Data_Sheet_2.docx]
